# Association of Contemporary Statin Pretreatment Intensity and LDL-C Levels on the Incidence of STEMI Presentation

**DOI:** 10.3390/life11111268

**Published:** 2021-11-19

**Authors:** Ziv Dadon, Mady Moriel, Zaza Iakobishvili, Elad Asher, Tal Y. Samuel, Dov Gavish, Michael Glikson, Shmuel Gottlieb

**Affiliations:** 1 Jesselson Integrated Heart Center, Shaare Zedek Medical Center, Jerusalem 9103102, Israel; ziv.dadon@mail.huji.ac.il (Z.D.); moriel@szmc.org.il (M.M.); easher@szmc.org.il (E.A.); talsamuel81@gmail.com (T.Y.S.); gavishd@szmc.org.il (D.G.); mglikson@szmc.org.il (M.G.); 2 “Clalit” Health Services, Tel-Aviv District, Tel Aviv-Yafo 6209804, Israel; zaza.iakobishvili@gmail.com; 3 Sackler Faculty of Medicine, Tel Aviv University, Ramat Aviv, Tel Aviv 69978, Israel; 4 Faculty of Medicine, Campus Ein Kerem, The Hebrew University, Jerusalem 9112102, Israel

**Keywords:** acute coronary syndrome, low-density lipoprotein cholesterol, myocardial infarction, primary prevention, secondary prevention, statin

## Abstract

Constituting hypolipidemic and pleiotropic effects, statins stabilize coronary artery plaque and may prevent STEMI events. This study investigated the association between contemporary statin pretreatment intensity, low-density lipoprotein cholesterol (LDL-C) levels, and the type of acute coronary syndrome (ACS) presentation: STEMI vs. NSTE-ACS. Data were drawn from the ACS Israeli Survey (ACSIS), a biennial prospective national survey that took place in 2008–2018. The rate of STEMI vs. NSTE-ACS was calculated by statin use, including statin intensity (high-intensity statin therapy (HIST) and low-intensity statin therapy (LIST) prior to the index ACS event. Among 5103 patients, 2839 (56%) were statin-naive, 1389 (27%) used LIST and 875 (17%) used HIST. Statin pretreated patients were older and had a higher rates of co-morbidities, cardiovascular disease history and pretreatment with evidence-based medications. STEMI vs. NSTE-ACS was lower among HIST vs. LIST vs. statin-naive patients (31.0%, 37.8%, and 54.0%, respectively, *p* for trend < 0.001). Multivariate analysis revealed that HIST was independently associated with lower STEMI presentation (OR_adj_ 0.70; 95% CI 0.57–0.86), while LIST (OR_adj_ 0.92; 95% CI 0.77–1.10) and LDL-C < 70 mg/dL (OR_adj_ 0.96; 95% CI 0.82–1.14) were not. In conclusion, among patients admitted with ACS, pretreatment with HIST was independently associated with a lower probability of STEMI presentation, while LIST and LDL-C < 70 mg/dL were not.

## 1. Introduction

Statins form the cornerstone for the prevention of acute coronary syndrome (ACS), with well-established beneficial use in the last few decades [1]. Indeed, both the 2018 American Heart Association/American College of Cardiology and the 2019 European Society of Cardiology (ESC) guidelines recommended that high-intensity statin therapy (HIST) should be the first-line treatment in all ACS patients, regardless of initial low-density lipoprotein cholesterol (LDL-C) values, and further treatment should be adjusted to reach predefined individualized LDL-C goals [2,3].

While LDL-C levels’ reduction is the main effect of statins, their pleiotropic effects may contribute to plaque stabilization, thus reducing the burden of vulnerable coronary plaque rupture, especially when ST-elevation myocardial infarction (STEMI) presentation is addressed [4,5]. Prior studies have demonstrated that the use of statins as primary or secondary prevention reduces STEMI prevalence among patients presenting with ACS [6,7,8,9,10]. These studies have not evaluated statin intensity nor the LDL-C level at presentation.

In this study, we sought to evaluate the association between contemporary statin treatment intensity (HIST vs. low-intensity statin therapy (LIST) prior to the index ACS event, LDL-C levels, and type of ACS at presentation (STEMI vs. non-ST-elevation ACS (NSTE-ACS), including non-STEMI and unstable angina pectoris (UAP) in a “real world” patient population during the last decade.

## 2. Materials and Methods

### 2.1. Study Population

This study consisted of data from the Acute Coronary Syndrome Israeli Survey (ACSIS) during the years 2008, 2010, 2016 and 2018 (the 2013 survey was excluded, due to lack of statin dosage documentation). ACSIS is a prospective nationwide community-based two-month biennial survey of all cases admitted with ACS in all 25 operating public Intensive Coronary Care Units/Cardiology Departments in Israel [11]. Demographic, historical, and clinical data; admission electrocardiography; pre-hospital medical therapies; lipid profile; and presenting characteristics were recorded.

The criteria for the diagnosis of the type of ACS were defined by the executive committee of the survey, according to clinical presentation, electrocardiographic findings, and cardiac biomarkers. The organization, data acquisition, management, and follow-up were performed at the Israeli Association for Cardiovascular Trials national coordinating center. The study protocol conforms to the ethical guidelines of the 1975 Declaration of Helsinki, as reflected in a priori approval by the institution’s human research committee. Data collection was approved at each hospital by the local institutional review board.

The patients were divided into 3 subgroups according to their chronic statin treatment status (as was documented individually upon admission and recorded in the predefined forms): HIST, LIST, and statin-naive patients. HIST was defined as a chronic daily dose of atorvastatin ≥40 mg, rosuvastatin ≥20 mg or simvastatin 80 mg [2]; and LIST as atorvastatin <40 mg, rosuvastatin <20 mg, or simvastatin ≤40 mg; patients not receiving statins were considered statin-naive.

The patients were further divided into five subgroups according to their LDL-C levels on admission: <55, 55–69, 70–100, 101–130, and >130 mg/dL (meaningful values according to recent ESC guidelines) [3,12].

### 2.2. Patients’ Inclusion/Exclusion/Withdrawal Criteria

Patients with known data regarding chronic statin treatment (more than a month prior to the index ACS event) and intensity, LDL-C levels, and an interpretable electrocardiogram at presentation were included in the study. The electrocardiogram interpretation was determined according to the diagnosis of the attending physician in each center. The lipid profile was obtained from blood samples drawn upon admission during the index ACS hospitalization.

### 2.3. Statistical Analyses

Patients’ characteristics according to chronic statin treatment groups (statin-naive, statin-treated, HIST, and LIST) were presented. Comparisons between statin-treated vs. statin-naive groups were tested, alongside HIST vs. LIST comparisons. Baseline characteristics with <15% missing values were imputed with baseline value “No”. The differences between the groups were tested with chi-square for categorical variables and with *t*-test or Mann–Whitney U test for continuous variables, where indicated. The association between statin intensity treatment and statin-naive on the proportion of patients presenting with STEMI vs. NSTE-ACS was analyzed among each of the subgroups. 

As significant differences were assumed between chronic statin therapy vs. statin-naive patients, a propensity score adjusting for statin use was calculated, using multivariate logistic regression analysis. The following variables were included: age, sex, weight, survey year, medical history of myocardial infarction (MI), CVA/TIA, coronary artery bypass grafting, percutaneous coronary intervention, angina pectoris, congestive heart failure, chronic kidney disease, PVD, dyslipidemia, hypertension, diabetes mellitus, family history of CAD, smoking and chronic medication treatment, including aspirin, clopidogrel, anticoagulants, β-blockers, angiotensin-converting enzyme inhibitors/angiotensin-II receptor blockers (ACE-I/ARB), spironolactone, fibrates, ezetimibe, calcium channel blockers and nitrates, and lipid profile on admission (total cholesterol, LDL-C, high-density lipoprotein cholesterol and triglycerides).

The association between prior statin use (HIST vs. LIST) and the proportion of patients presenting with STEMI vs. NSTE-ACS was analyzed by multivariate logistic regression analysis with STEMI at presentation as the dependent variable, and the covariates were propensity score for statin use (see above), statin treatment status (LIST, HIST and naive; reference group statin-naive patients), LDL-C < 70 mg/dL, with and without prior relevant medication use (aspirin, clopidogrel, β-blockers and ACE-I/ARB). Odds ratio (OR) and 95% confidence interval (95% CI) were calculated for each covariate.

Statistical analyses were performed using R Core Team software (2020, version-4.0.0, Vienna, Austria). All tests were two-sided and a *p* value < 0.05 was considered statistically significant.

## 3. Results

A total of 5424 ACS patients were included in the four surveys (2008, 2010, 2016, and 2018). Cases with missing data regarding statin intensity (*n* = 321) were excluded from the study, leaving 5103 patients who met the inclusion criteria for analysis.

### 3.1. Baseline Characteristics

Statin-treated vs. statin-naive: Among the 5103 patients, 2839 (55.6%) were statin-naive, and 2264 (44.4%) were treated with statins (Table 1). As compared to statin-naive patients, statin-treated patients were older, more often women, more likely to suffer from comorbidities and CVD, more often received evidence-based medications prior to index hospitalization, and had worse Killip class at presentation and higher blood pressure. As compared with statin-naive patients, statin-treated patients had a better lipid profile.

HIST vs. LIST: Among 2264 statin-treated patients, 875 (38.6%) were HIST and 1389 (61.4%) were LIST patients (Table 1). The proportion of HIST patients increased from 19% in 2008–2010 to 69% in 2016–2018. As compared with LIST patients, HIST patients were younger, less often women, more likely to suffer from comorbidities and CVD, and be treated with evidence-based medications prior to the index hospitalization. HIST patients had similar levels of entire lipid profile as compare with LIST patients (aside from total cholesterol).

### 3.2. Association between Statin Therapy, LDL-C Levels and ACS Type

**Figure 1:**
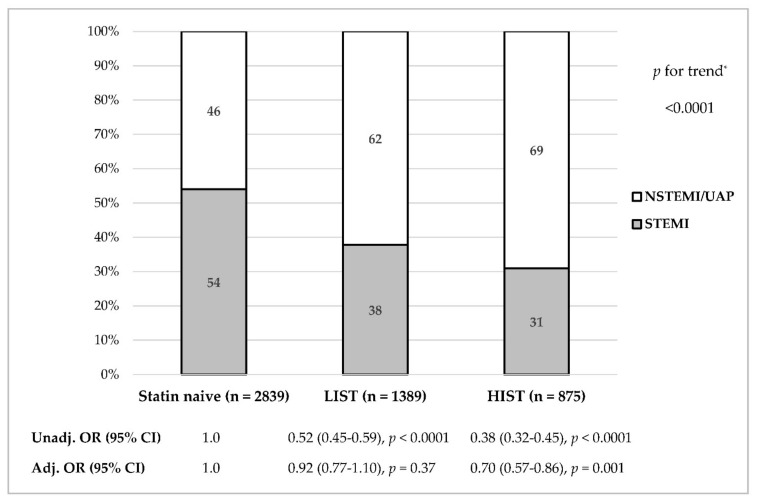
Rates of STEMI vs. NSTE-ACS according to statin pretreatment intensity. The figure presents the rates of STEMI vs. NSTE-ACS among patients presenting with ACS according to statin treatment prior to index event: HIST, LIST and statin-naive patients. Unadjusted and multivariate-adjusted OR with 95% CI for STEMI presentation are shown for each group (reference group naive patients). * *p* value for the comparison between statin-naive vs. LIST was <0.0001 and for the comparison between LIST vs. HIST was 0.001. *Abbreviations*: ACS, acute coronary syndrome; Adj, adjusted; CI, confidence interval; HIST, high-intensity statin therapy; LIST, low-intensity statin therapy; NSTE-ACS, non-ST elevation acute coronary syndrome; OR, odds ratio; STEMI, ST-elevation myocardial infarction; Unadj, unadjusted.

**Figure 2:**
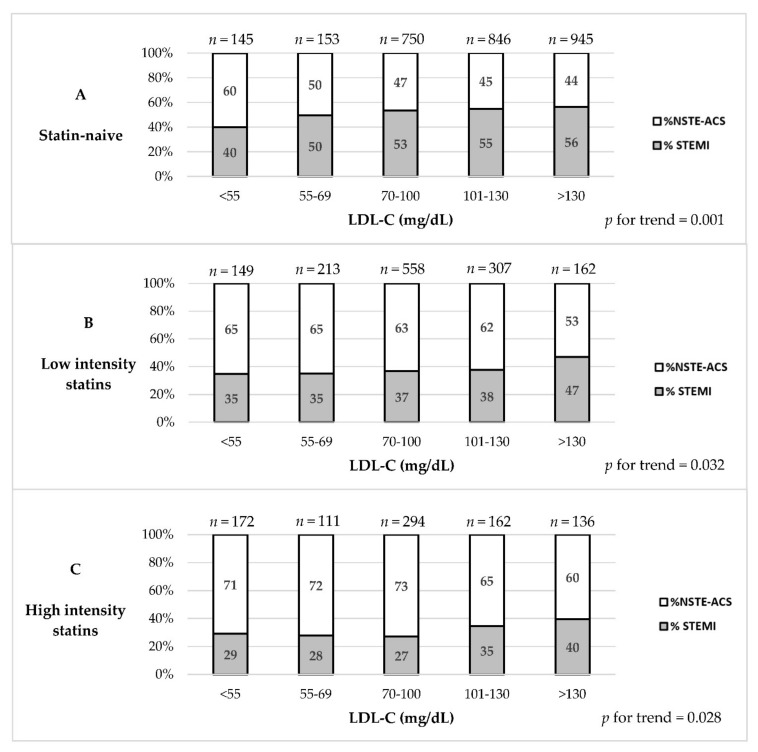
Rates of STEMI vs. NSTE-ACS according to LDL-C levels and statin pretreatment intensity among patients presenting with ACS. The figure shows the rates of STEMI vs. NSTE-ACS according to statin pretreatment intensity and at 5 LDL-C levels, among patients presenting with ACS. Statin pretreatment status included: (**A**) statin-naive patients; (**B**) low-intensity statin therapy (LIST); (**C**) high-intensity statin therapy (HIST). *Abbreviations*. As in Figure 1; LDL-C, low-density lipoprotein cholesterol; mg/dL, milligrams per deciliter.

The proportion of patients presenting with STEMI vs. NSTE-ACS was significantly lower among HIST as compared with LIST and statin-naive patients (31.0% vs. 37.8% vs. 54.0%, respectively; *p* < 0.001, Figure 1). As shown in Figure 2 and Table 2, at each LDL-C level, the proportion of STEMI was the lowest among HIST patients (*p* for trend < 0.001). With the decline in LDL-C level, the proportion of STEMI decreased among the HIST, LIST and statin-naive patients (*p* for trend 0.028, 0.032, and 0.001, respectively). A lower proportion of STEMI was also observed among patients with LDL-C < 70 mg/dL when comparing HIST and LIST vs. statin-naive patients (28.6% vs. 35.1% vs. 45.0%, respectively; *p* < 0.001). Furthermore, HIST patients with the highest LDL-C level (>130 mg/dL) presented with STEMI as frequently as statin-naive patients with the lowest LDL-C level (<55 mg/dL; 40% in both groups).

Consistent results were obtained when the analyses were carried out separately among patients with or without known CAD; HIST as compared to LIST and statin-naive patients presented less often with STEMI (28%, 32%, and 41%, *p* < 0.001; 40%, 45%, and 57%, *p* < 0.001, respectively; data not shown). Similarly, among patients with or without known prior CVD (CAD, PVD or CVA/TIA), HIST as compared to LIST and statin-naive patients presented less often with STEMI (28%, 33%, and 42%, *p* < 0.001; 43%, 46%, and 58%, *p* < 0.001, respectively; data not shown).

### 3.3. Association between Statin and Ezetimibe Therapy and ACS Type

Ezetimibe was used only among 103 patients: 56 combined with HIST, 27 combined with LIST, and 20 without. There was no difference in the presentation of STEMI vs. NSTE-ACS, neither in the ezetimibe–statin nor in the ezetimibe–statin-naive group.

### 3.4. Multivariate Analyses for STEMI Presentation

As shown in Table 3 and Figure 1, multivariate analysis adjusting for the propensity score for chronic statin treatment, including pertinent variables (see Statistical Analyses), revealed that the higher the propensity score was, the lower the probability of presenting with STEMI (Model 1). Adding statin intensity sub-groups and LDL-C < 70 mg/dL as covariates into Model 1 (Model 2) revealed that only HIST was independently associated with lower probability for STEMI presentation (OR_adj_ = 0.70; 95% CI 0.57–0.86, *p* = 0.001), while LIST and LDL-C < 70 mg/dL were not (OR_adj_ = 0.92, 95% CI 0.77–1.10, *p* = 0.37; OR_adj_ = 0.96; 95% CI 0.82–1.14, *p* = 0.97, respectively). A separate analysis adding other evidence-based medications into Model 2 (Model 3) revealed similar results: only HIST remained a significant predictor for STEMI presentation, while LIST and LDL < 70 mg/dL were not.

## 4. Discussion

The current study evaluated the association between contemporary chronic statin treatment intensity (HIST vs. LIST) prior to the index ACS event, LDL-C level, and the type of ACS presentation (STEMI vs. NSTE-ACS) in a “real world” patient population, during 2008 to 2018. Both chronic HIST and LIST groups were associated with a lower proportion of STEMI presentation as compared with statin-naive patients (31% vs. 38% vs. 54%, respectively). Nonetheless, after adjustment for propensity score and potential pertinent confounders, only HIST was associated with a lower proportion of STEMI presentation, whereas LIST and LDL-C level < 70 mg/dL were not. The benefit of HIST was also demonstrated in patients with and without prior CAD or CVD.

### 4.1. Prior Studies

Several prior studies have demonstrated that, among patients presenting with ACS, pretreatment with statins was associated with a lower incidence of STEMI [6,7,8,9,10]. These studies included data from the previous decade where the use of HIST was less frequent. Furthermore, none of these studies, including a recent publication [13], have evaluated statin pretreatment intensity nor the association between LDL-C level and STEMI presentation. In that study [13], the proportion of patients on statin pretreatment was low (18%) and the incidence of STEMI with and without statin pretreatment was 52% and 64%, respectively, higher figures than in our study.

In a previous study, we have demonstrated that among patients with ACS admitted during 2002–2010, statin use but not LDL-C level < 70 mg/dL was associated with a lower probability of STEMI presentation [14]. We have also evaluated statin pretreatment intensity, demonstrating that patients on HIST had the lowest likelihood of presenting with STEMI. However, as it was not emphasized by previous guidelines published in the prior decade, the proportion of HIST among statin-treated patients was relatively low (19%). In the present study, conducted in recent years (2008–2018), where the proportion of HIST patients doubled (39%), we have demonstrated that only HIST pretreatment was associated with a lower rate of STEMI presentation, whereas pretreatment with LIST was not.

### 4.2. Statin Intensity and Plaque Characteristics

Substantial experimental and clinical studies have demonstrated that statins stabilize coronary plaques not only through LDL-C levels’ reduction but via numerous mechanisms, namely pleiotropic effects, thereby reducing the risk of plaque rupture, the leading mechanism causing coronary occlusion and a subsequent STEMI event [15]. New imaging modalities enable the assessment of plaque characteristics and local inflammatory processes, and thus, may support our study findings.

In a clinical trial using intravascular ultrasound, chronic HIST reduced coronary artery plaque burden [16]. Furthermore, LIST was shown to be associated with atherosclerosis progression while HIST was not [17].

Optical coherence tomography (OCT) can assess plaque characteristics including thin-cap fibroatheroma, a key component in plaque vulnerability and the pathophysiology of STEMI [18]. Statin pretreatment reduced ruptured-plaque and thin-cap fibroatheroma rates in STEMI [18] and increased fibrous cap thickness independent of coronary risk factors [19]. Moreover, the prevalence of thin-cap fibroatheroma and active inflammation process was significantly lower in HIST as compared to LIST [20].

As compared with LIST, HIST elicited a greater reduction in carotid and aortic plaque fluorodeoxyglucose uptake during PET-computerized tomography (may represent a lower plaque inflammation) [21]. The uptake reduction did not correlate with lipid profile changes, suggesting that HIST has an additional independent effect on plaque features. These findings propose an additional physiological mechanism to statin treatment.

Coronary artery calcification (CAC) represents an advanced stage of atherosclerosis, and a stabilization force in high-risk plaques [22]. HIST was associated with atheroma volume regression among patients with known CAD, whereas both LIST and statin-naive treatment were associated with percent atheroma volume progression [23]. The greatest increase in CAC indices was noted among HIST patients. Another study demonstrated that among patients enrolled to HIST and LIST arms, an increase in CAC volume was only noted in the HIST group [24].

These studies provide insight as to the way statins, particularly HIST, may lead to plaque stabilization, beyond their effect on the regression of the plaque. These physiological effects of statins may explain our observation that only HIST was associated with a lower probability of STEMI presentation, whereas LIST was not.

### 4.3. STEMI Presentation in Reduced LDL-C Levels Settings

There are a lack of data regarding the impact of low LDL-C levels on STEMI rate in the setting of statin monotherapy. In a recent publication studying the effects of the proprotein convertase subtilisin/kexin type 9 (PCSK9) inhibitor, evolocumab, when added to maximally tolerated statin dose or a high dose, among patients with atherosclerotic cardiovascular disease, LDL-C levels were reduced from 92 mg/dL to 30 mg/dL [25]. Evolocumab use was associated with a subsequent decline in ACS events, with a greater risk reduction in STEMI vs. NSTE-MI (36% vs. 23%, respectively) [26]. Similar findings were observed in the ODYSSEY OUTCOMES trial using alirocumab added to high-dose statins, among patients after ACS. STEMI incidence was lower than NSTE-ACS in the follow-up (0.5% vs. 4.8%, respectively) [27]. These findings can be further understood considering the recent HUYGENS Phase III trial results, demonstrating by serial OCT imaging, a benefit of adding evolocumab to statin therapy among NSTE-ACS patients by increasing fibrous cap thickness [28].

In our study, we have demonstrated that with the decline in LDL-C levels, the proportion of STEMI rates decreased among each of the statin subgroups. However, despite pretreatment with HIST, 29% of the patients with LDL-C < 55 mg/dL presented with STEMI. The use of ezetimibe was observed only in a few patients and none received PCSK9 inhibitors. These findings suggest that the recommended management for ACS prevention should include the maximally tolerated statin dose along with the combination of other lipid-lowering agents with ezetimibe and/or PCSK9 inhibitors to aim for the lowest achievable LDL-C levels.

There is growing evidence that inflammation plays an important role in atherosclerosis pathophysiology, especially among STEMI patients [29]. Recent studies have shown the benefit of anti-inflammatory drugs, including interleukin-1β inhibitors and colchicine [30,31]. It is conceivable that targeting both the lipid profile and the inflammatory process may contribute to a lower STEMI presentation, mainly secondary to plaque stabilization improvement.

### 4.4. HIST in Primary and Secondary Prevention

Our results have demonstrated that HIST was associated with a lower STEMI incidence among both patients with and without prior CAD or CVD. These findings highlight that HIST should be prescribed up to the highest tolerated dose to reach the goals set for the specific level of risk for both primary and secondary prevention, as being emphasized in the current guidelines [2,3].

### 4.5. Limitations

Our study is a multicenter, prospective, observational, nationwide survey and not a randomized trial, and as such, is subjected to confounding factors. To minimize confounding for statin pretreatment, we used the propensity score method that was included in the multivariate analysis models. Only patients with known data regarding statin dosages were included in the trial; however, statin pretreatment was defined as treatment that was received for at least one month prior to the index ACS event. There are a lack of data on the duration of pretreatment, which may cause underestimation of statin advantages. Nonetheless, the study provides contemporary real-world data.

## 5. Conclusions

Among patients admitted with ACS, pretreatment with high dose statins was independently associated with a lower probability of STEMI presentation, while low-dose statins and LDL-C < 70 mg/dL were not. Our study underscores the stabilizing effect of statins on the atheromatous plaque as supported by the newly emerged imaging modalities, and highlight the current guidelines’ recommendations, emphasizing the importance of the highest tolerated statin dose as the first-line therapy to reach the goals set for the specific level of risk for both primary and secondary prevention. Future research using combination therapies of statins along with ezetimibe and/or PCSK9 inhibitors and anti-inflammatory drugs may answer whether such combinations would lead to a further reduction in STEMI incidence.

## Figures and Tables

**Table 1:** Baseline characteristics by chronic statin treatment and intensity.

Variable	Statin-Naive *n* = 2839	Statin-Treated *n* = 2264	*p*-Value ^1^	Low Intensity *n* = 1389	High Intensity *n* = 875	*p*-Value ^2^
**Patients’ characteristics**						
Age, years (mean ± SD)	61.4 ± 13.15	66.1 ± 11.7	<0.001	67.0 ± 11.9	64.8 ± 11.4	<0.001
Female sex, *n* (%)	508 (17.9)	546 (24.1)	<0.001	372 (26.8)	174 (19.9)	<0.001
**Medical history**						
Diabetes mellitus, *n* (%)	766 (27.0)	1142 (50.4)	<0.001	655 (47.2)	487 (55.7)	<0.001
Hypertension, *n* (%)	1412 (49.7)	1761 (77.8)	<0.001	1052 (75.7)	709 (81.0)	0.004
Current smoker, *n* (%)	1334 (47.0)	712 (31.4)	<0.001	397 (28.6)	315 (36.0)	<0.001
CAD family history, *n* (%)	800 (28.2)	590 (26.1)	0.097	339 (24.4)	251 (28.7)	0.027
Dyslipidemia, *n* (%)	1522 (53.6)	2139 (94.5)	<0.001	1322 (95.2)	817 (93.4)	0.082
CKD, *n* (%)	202 (7.1)	373 (16.5)	<0.001	210 (15.1)	163 (18.6)	<0.001
Prior MI, *n* (%)	486 (17.1)	1185 (52.3)	<0.001	606 (43.6)	579 (66.2)	<0.001
Past PCI/CABG, *n* (%)	464 (16.3)	1302 (57.5)	<0.001	690 (49.7)	612 (69.9)	<0.001
Past CVA/TIA, *n* (%)	160 (5.6)	251 (11.1)	<0.001	144 (10.4)	107 (12.2)	0.192
PVD, *n* (%)	139 (4.9)	246 (10.8)	<0.001	137 (9.9)	109 (12.5)	0.063
**Chronic medical treatment**						
ACE-I/ARB, *n* (%)	617 (21.7)	1380 (61.0)	<0.001	788 (56.7)	592 (67.7)	<0.001
Aspirin, *n* (%)	598 (21.1)	1687 (74.5)	<0.001	981 (70.6)	706 (80.7)	<0.001
Clopidogrel, *n* (%)	85 (3.0)	461 (20.4)	<0.001	213 (15.3)	248 (28.3)	<0.001
Anti-platelets, *n* (%)	99 (3.5)	528 (23.3)	<0.001	220 (15.8)	308 (35.2)	<0.001
Ezetimibe, *n* (%)	20 (0.7)	83 (3.7)	<0.001	27 (1.9)	56 (6.4)	<0.001
Beta blockers, *n* (%)	454 (16.0)	1300 (57.4)	<0.001	741 (53.3)	559 (63.9)	<0.001
**Presenting characteristics**						
STEMI diagnosis, *n* (%)	1533 (54.0)	796 (35.2)	<0.001	525 (37.8)	271 (31.0)	0.001
Killip class > I, *n* (%)	267 (9.4)	290 (12.8)	0.001	180 (13.0)	110 (12.6)	0.838
HR (bpm), median (IQR)	78 (68, 90)	78 (67, 90)	0.602	77 (66, 90)	79 (68, 91)	0.057
SBP (mmHg), median (IQR)	140 (123, 159)	140 (124, 160)	0.044	140 (124, 160)	141 (125, 160)	0.365
**Lipid profile**						
TC (mg/dL), median (IQR)	181 (153, 211)	155 (131, 183)	<0.001	157 (134, 184)	151 (126, 182)	0.027
LDL (mg/dL), median (IQR)	113 (89, 139)	86 (67, 110)	<0.001	88 (68, 109)	84 (63, 112)	0.400
HDL (mg/dL), median (IQR)	38 (32, 46)	38 (32, 46)	0.610	39 (32, 47)	37 (30, 44)	0.230
TG (mg/dL), median (IQR)	124 (89, 176)	129 (91, 185)	0.005	128 (89, 181)	130 (95, 188)	0.162

^1^ Statin-naive vs. statin-treated groups. ^2^ Low-intensity statin therapy vs. high-intensity statin therapy. *Abbreviations*: ACE-I, angiotensin-converting enzyme inhibitor; ARB, angiotensin-II receptor blocker; bpm, beats-per-minute; CABG, coronary artery bypass grafting; CAD, coronary artery disease; CKD, chronic kidney disease; CVA, cerebrovascular accident; ECG, electrocardiogram; HDL-C; high-density lipoprotein cholesterol; HR, heart rate; IQR, interquartile range; LDL-C, low-density lipoprotein cholesterol; mg/dL, milligrams per deciliter; MI, myocardial infarction; mmHg, millimeters of Mercury; *n*, number; PCI, percutaneous coronary intervention; PVD, peripheral vascular disease; SBP, systolic blood pressure; SD, standard deviation; STEMI, ST-elevation myocardial infarction; TC, total cholesterol; TG, triglycerides; TIA, transient ischemic attack; UAP, unstable angina pectoris.

**Table 2:** STEMI vs. NSTE-ACS according to use and intensity of statin treatment prior to index event as divided by five different LDL-C level subgroups.

LDL-C Subgroups (mg/dL)	<55	55–69	70–99	100–129	>130	All	*p* for Trend
**A. All patients**							
*n*	466	477	1602	1315	1243	5103	
STEMI rates, *n* (%)	160 (34.3)	182 (38.2)	687 (42.9)	637 (48.4)	663 (53.3)	2329 (45.6)	<0.001
**B. Statin naive patients**							
*n*	145	153	750	846	945	2839	
STEMI rates, *n* (%)	58 (40.0)	76 (49.7)	401 (53.5)	465 (55.0)	533 (56.4)	1533 (54.0)	0.001
**C. Statin treated patients**							
*n*	321	324	852	469	298	2264	
STEMI rates, *n* (%)	102 (31.8)	106 (32.7)	286 (33.6)	172 (36.7)	130 (43.6)	796 (35.2)	0.001
**D. Low-intensity statin treated patients**							
*n*	149	213	558	307	162	1389	0.032
STEMI rates, *n* (%)	52 (34.9)	75 (35.2)	206 (36.9)	116 (37.8)	76 (46.9)	525 (37.8)	
**E. High-intensity statin treated patients**							
*n*	172	111	294	162	136	875	
STEMI rates, *n* (%)	50 (29.1)	31 (27.9)	80 (27.2)	56 (34.6)	54 (39.7)	271 (31.0)	0.028

*Abbreviations*: LDL-C, low-density lipoprotein cholesterol; mg/dL, milligrams per deciliter; *n*, number; NSTE-ACS, non-ST-elevation acute coronary syndrome; STEMI, ST-elevation myocardial infarction; UAP, unstable angina pectoris; vs., versus.

**Table 3:** Predictors of STEMI presentation.

Variable	Multivariate Analysis			
	OR ^a^	95% CI	*p* Value	c-Statistics
**Model 1:** **Propensity score quintiles**				0.632
1 (reference)	1.0			
2 (vs. 1st quintile)	0.88	0.74–1.06	0.65	
3 (vs. 1st quintile)	0.65	0.54–0.78	<0.001	
4 (vs. 1st quintile)	0.42	0.35–0.51	<0.001	
5 (vs. 1st quintile)	0.26	0.21–0.31	<0.001	
**Model 2:**				0.635
Propensity score	0.31	0.24–0.39	<0.001	
Low-intensity statins (vs. statin-naive)	0.92	0.77–1.10	0.37	
High-intensity statins (vs. statin-naive)	0.70	0.57–0.86	0.001	
LDL-C < 70 mg/dL (vs. ≥70)	0.96	0.82–1.14	0.67	
**Model 3 ^b^:**				0.648
Propensity score	0.66	0.45–0.96	0.03	
Low-intensity statins (vs. statin-naive)	0.88	0.74–1.05	0.17	
High-intensity statins (vs. statin-naive)	0.75	0.61–0.93	0.01	
LDL-C < 70 mg/dL (vs. ≥70)	0.94	0.80–1.11	0.49	
Chronic aspirin (yes vs. no)	0.92	0.76–1.10	0.36	
Chronic clopidogrel (yes vs. no)	0.58	0.46–0.73	<0.001	
Chronic ACE-I/ARB (yes vs. no)	0.77	0.66–0.89	<0.001	
Chronic Beta-blocker (yes vs. no)	0.71	0.63–0.83	<0.001	

Propensity score indicated the likelihood of statin treatment prior to index ACS event. Propensity score was calculated by logistic regression analysis including 31 covariates (see “statistical analyses”). The quintiles used for propensity score included: 0–0.2 (reference group), 0.2–0.4, 0.4–0.6, 0.6–0.8, and 0.8–1.0. ^a^ The OR was calculated by logistic regression analysis adjusting for: Model 1, the propensity score alone; Model 2, the propensity score with the variables prior statin use and intensity and LDL < 70 mg/dL; Model 3, variables as in Model 2 along with the variables: prior use of clopidogrel, aspirin, β-blockers and ACE-I/ARB (see “Methods”). ^b^ Similar results were obtained entering the medications into the model separately, one at a time. Abbreviations as in Table 1; CI, confidence interval.

## Data Availability

Data sharing not applicable.
